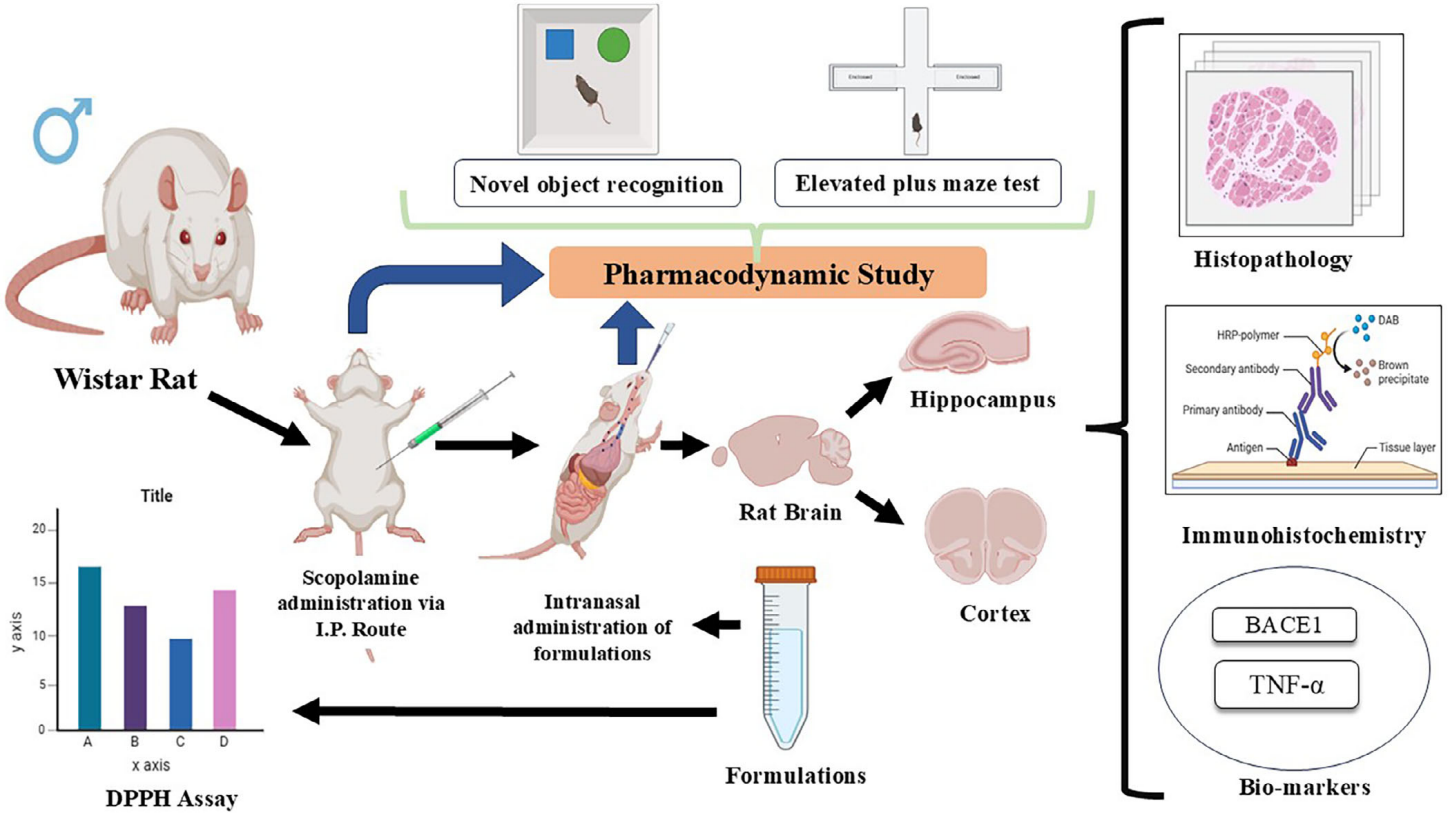# Innovative Intranasal Nanocarriers of Butyrylcholinesterase and Kinase Inhibitors for Alzheimer’s Disease: Immunohistochemistry, Biomarkers, Histopathology, and Antioxidant Perspective

**DOI:** 10.1002/alz70859_102657

**Published:** 2025-12-25

**Authors:** Saif Ahmad Khan, Zufika Qamar, Neha Bhardwaj, Ashif Iqubal, Pirthi Pal Singh, Suhel Pervez, Sanjula Baboota, Javed Ali

**Affiliations:** ^1^ Jamia Hamdard, New Delhi, Delhi India; ^2^ SGT University, Gurugram, Haryana India; ^3^ Jamia Hamdard, Hamdard Ngar, New Delhi India; ^4^ Tirupati Group, Paonta Sahib, Himachal Pradesh India; ^5^ Jamia Hamdard, New Delhi, New Delhi India; ^6^ Jamia Hamdard, New Delhi, New Delhi India

## Abstract

**Background:**

Alzheimer’s disease (AD) is progressive neurodegenerative disorder characterized by cognitive decline, oxidative stress, and limited therapeutic options to address both pathology and oxidative damage. Intranasal delivery of nanostructured lipid carriers (NLCs) offers promising approach to enhance brain bioavailability of drugs. This study investigates NLCs loaded with Butyrylcholinesterase (RV) and Kinase Inhibitors (KI) for targeted delivery in AD therapy. Using scopolamine‐induced AD‐like symptoms model in male Wistar rats, pharmacokinetic, biodistribution, and pharmacodynamic studies, including Novel Object Recognition (NOR) and Elevated Plus Maze (EPM) tests, were conducted alongside immunohistochemistry (IHC), histopathology, and antioxidant assays to evaluate therapeutic efficacy of RV‐KI‐NLCs.

**Method:**

The study aimed to enhance the brain bioavailability of RV and KI using NLCs for intranasal delivery in Alzheimer’s disease. NLCs, prepared via a modified emulsification method, were characterized and evaluated in scopolamine‐induced AD model rats through pharmacokinetic, biodistribution, and pharmacodynamic studies, including NOR and EPM tests. Immunohistochemistry and histopathological analyses assessed AD biomarkers (BACE1, TNF‐α), neuronal integrity, and inflammation, while the DPPH assay confirmed antioxidant potential. RV‐KI‐NLCs demonstrated significant efficacy in reversing scopolamine‐induced AD‐like symptoms.

**Result:**

Histopathological analysis of hippocampus (CA1) and cortex regions revealed significant neurotoxicity, including cellular disintegration, pyknosis, and vacuolation in the scopolamine‐treated negative control group, while no cellular damage was observed in positive control group. Among treatments, RV‐KI‐NLC formulation exhibited the highest neuroprotective efficacy, followed by KI‐NLC and RV‐NLC, with all NLC‐treated groups significantly mitigating scopolamine‐induced damage compared to suspension‐treated groups. RV‐KI suspension showed moderate improvement, outperforming RV and KI suspensions. Immunohistochemistry demonstrated marked reduction in BACE1, and TNF‐α in the RV‐KI‐NLC‐treated group, accompanied by improved neuronal integrity, reduced gliosis, and lower neuroinflammation. DPPH assay confirmed the high antioxidant activity of RV‐KI‐NLC, indicating strong free radical scavenging potential. These findings aligned with behavioral improvements observed in NOR and EPM tests, highlighting formulation’s ability to effectively restore cognitive and behavioral functions.

**Conclusion:**

The RV‐KI‐NLC formulation showed superior neuroprotective effects in scopolamine‐induced AD‐like symptoms, with significant improvements in neuronal integrity, reduced neuroinflammation, enhanced cognitive function, and strong antioxidant activity, highlighting its potential as a promising therapeutic strategy for AD.